# Shaping tomorrow’s dentists: a multi-institutional survey of undergraduate dental students’ perceptions towards interprofessional education

**DOI:** 10.1186/s12903-024-04532-y

**Published:** 2024-07-04

**Authors:** Galvin Sim Siang Lin, Yook Shiang Ng, Hasnah Hashim, Chan Choong Foong, Noor Azlin Yahya, Mohd Haikal Muhamad Halil, Mas Suryalis Ahmad

**Affiliations:** 1 Department of Restorative Dentistry, Kulliyyah of Dentistry, International Islamic University Malaysia, Kuantan Campus, Kuantan, Pahang 25200 Malaysia; 2 Department of Dental Materials, Faculty of Dentistry, Asian Institute of Medicine, Science and Technology (AIMST) University, Bedong, Kedah 08100 Malaysia; 3 Department of Dental Public Health, Faculty of Dentistry, Asian Institute of Medicine, Science and Technology (AIMST) University, Bedong, Kedah 08100 Malaysia; 4 Medical Education and Research Development Unit (MERDU), Faculty of Medicine, Universiti Malaya, Kuala Lumpur, 50603 Malaysia; 5 Department of Restorative Dentistry, Faculty of Dentistry, Universiti Malaya, Kuala Lumpur, 50603 Malaysia; 6 Centre of Comprehensive Care Dentistry, Faculty of Dentistry, Universiti Teknologi MARA, Sungai Buloh Campus, Shah Alam, Selangor 47000 Malaysia

**Keywords:** Collaborative learning, Dentistry, Health profession, Interprofessional education, Undergraduate

## Abstract

**Background:**

Interprofessional education (IPE) is essential to foster collaboration among healthcare professionals for holistic patient care. However, Malaysian dental education remains discipline-centric, hindering multidisciplinary learning approaches. Hence, this study aimed to explore Malaysian undergraduate dental students’ perceptions of IPE.

**Methods:**

The present cross-sectional study employed convenience sampling to survey undergraduate dental students from four Malaysian institutions using a modified questionnaire with 20 close-ended and 2 open-ended questions. The questionnaire covered three domains (effectiveness, preference, importance) to assess students’ perceptions using a five-point Likert scale. Psychometric validation was performed to assure validity and reliability of the modified questionnaire. Quantitative analysis (descriptive and inferential statistics), and qualitative analysis (content analysis) were subsequently performed.

**Results:**

397 students responded, and positive perceptions were generally noted with mean scores ranging from 4.13 to 4.35 across all domains. Questions 2 and 3, assessing the improvement in understanding the roles and responsibilities, and communication among healthcare professionals, received the highest mean scores. Meanwhile, Question 15 concerning the incorporation of IPE into educational goals received the lowest mean score. Regression analysis identified gender and clinical phase as significant factors, with females and preclinical students exhibiting more favourable perceptions. Motivators for IPE included a keen interest in diverse perspectives and recognising the importance of teamwork, while barriers encompassed tightly packed schedules, lack of understanding about IPE, misconceptions regarding dental education, and students’ nervousness and fear of participation.

**Conclusion:**

This study produced a valid and reliable instrument to measure undergraduate dental students’ perceptions towards IPE. Strategic planning, such as overcoming logistical challenges, improving awareness, and creating a supportive learning environment are crucial for successful IPE integration into existing curricula, especially in resource-constrained developing countries like Malaysia.

## Background

Interprofessional education (IPE) in healthcare represents a dynamic and evolving approach to learning that fosters collaboration among healthcare professionals from diverse disciplines, aiming to deliver comprehensive and holistic patient care [1]. Central to this concept is the cultivation of a collaborative mindset, mutual respect, a nuanced understanding of each discipline’s roles and responsibilities, and an appreciation of the unique contributions made by each professional group to patient well-being [2]. In this manner, each profession can take part in patient care issues that fall within the scope of their specialisation, and it is valued when decisions are made together. Moreover, IPE facilitates effective collaborative practice in healthcare which enhances patient outcomes and reduces global health workforce issues [3]. IPE has therefore emerged as a key element in the debate of the competencies of healthcare professionals and high-quality healthcare education, as healthcare providers should receive interprofessional training that is integrated into the curriculum to become competent and quality-focused professionals who are ready to work in a team [2, 4].

It is not surprising that universities worldwide have been challenged to develop and sustain authentic IPE activities that cover every aspect of their curricula [4, 5]. The Interprofessional Education Collaboration (IPEC) established in 2011 laid down fundamental competencies for IPE, offering objective benchmarks for evaluating IPE activities [6]. The four core competency domains are: (1). values and ethics; (2). roles and responsibilities for collaborative practice; (3). interprofessional communication; and (4). teamwork and team-based care. The goal of this competency set is to prepare students for lifetime learning and collaboration to enhance individual and patient care as well as population health outcomes. Nonetheless, medical and health professions education in Malaysia remains predominantly uni-professional and discipline-centric in nature [7]. This has led to the fragmentation of patient care, particularly in the management of complicated health conditions and chronic illnesses that require multidisciplinary approaches. Similarly, most Malaysian dental programs have historically placed little emphasis on interprofessional collaboration between dental students and students from other healthcare professions. In the past, Malaysian undergraduate dental students were required to complete fundamental medical sciences courses alongside medical students during their preclinical years. However, such collaborative learning activities have dwindled in recent years as dental students learn these courses at their respective faculties or schools in silos. With the growing recognition of the intricate relationship between oral health and overall health, and the necessity for oral health professionals to collaborate with other healthcare providers [8, 9], IPE is imperative to bridge these gaps and foster collaborative learning between dental students and their counterparts from other health professions [10].

Interprofessional education holds particular importance for dental practitioners because it enables them to work collaboratively with other healthcare professionals, including dental technologists, medical physicians, nurses, and pharmacists, to enhance patient care [10, 11]. By participating in IPE activities, dental practitioners can learn to communicate effectively, share knowledge and skills, and build relationships with other healthcare professionals. This improves the quality of care and outcomes for patients, particularly those with complex dental and medical issues [12]. IPE can also empower dental practitioners to develop skills such as leadership, teamwork, and problem-solving, which are essential in today’s healthcare environment [13]. Furthermore, IPE helps dental practitioners understand the importance of not only treating the oral health needs of their patients but also attending to their overall health needs [14]. The current body of literature underscores the critical need for dental education to transition from siloed practice to a collaborative team-based approach.

A recent study published in 2022 evaluating the perceptions of clinical healthcare students at a Malaysian institution towards IPE, indicated their support for IPE implementation [15]. Although IPE and collaborative practice are still gaining popularity globally, it is unclear whether Malaysian dental students would embrace IPE. Thus, strategic planning is necessary while transitioning from traditional education to IPE in dental curricula, particularly in resource-constrained developing countries [7]. To make informed decisions about the integration of the IPE approach into dental curricula across the nation, persuasive data is required, specifically regarding dental students’ perceptions. These perceptions offer valuable insights into the effectiveness of IPE initiatives, help identify potential barriers, and inform strategies to enhance the IPE experience for dental students. Therefore, the present study aimed is to determine Malaysian undergraduate dental students’ perceptions of interprofessional education.

## Methods

### Theoretical framework

The theoretical framework underpinning this study is rooted in social constructivism, which posits that learning is a collaborative and dynamic process shaped by interactions with others and the environment. Social constructivism, proposed by Lev Vygotsky, a Soviet psychologist in the post-revolutionary era, hypothesised that cognitive functions find their roots in social interactions [16]. Vygotsky argued that the explanation for cognitive functions lies in understanding them as products of these interactions. He challenged the notion that learning is merely the absorption and adjustment of new knowledge by learners; instead, he asserted that learning is a dynamic process where learners become integrated into a community of knowledge. In the context of IPE for healthcare professionals, social constructivism aligns with the idea that knowledge and skills are co-constructed through shared experiences and communication among individuals from different healthcare disciplines. The central tenet of social constructivism in IPE is the importance of collaboration, mutual respect, and understanding of diverse perspectives to foster effective learning among all healthcare professionals.

### Sampling and participants

The present study was conducted among undergraduate preclinical and clinical dental students from four different dental training institutions in Malaysia. Ethical approval was granted by the local university human ethics committee under the ethical approval code of AUHEC/FOD/2023/17. Raosoft^®^ Sample Size Calculator software (Raosoft Inc., Seattle, Washington, USA) was used to determine the required sample size. The total population of undergraduate dental students across the four dental training institutions was estimated to be approximately 1100 students. With a 5% margin of error and a 95% confidence interval, a minimum sample size of 285 respondents was determined to be necessary. Accounting for an anticipated non-response rate of 20%, the overall minimum sample size for the present study was set at 342 respondents.

### Design and setting

Convenience sampling was used to recruit undergraduate dental students currently enrolled in either the Bachelor of Dental Surgery (BDS) or Doctor of Dental Surgery (DDS) programs at four different dental training institutions. In Malaysia, both the BDS and DDS are five-year undergraduate dental programs. These programs are divided into two phases: the preclinical phase, which encompasses the first two years, and the clinical phase, which spans from the third to the fifth year. An online questionnaire was employed to assess the perceptions of undergraduate dental students’ perceptions regarding the effectiveness, preference, and significance of IPE in dental curricula. The online survey was prepared using Google Forms and shared with the students through WhatsApp groups. Students voluntarily and anonymously participated in the online survey after granting verbal and electronic written consent. They were allotted a two-week window to complete the questionnaire. A reminder was sent to the students after two weeks to complete the questionnaire.

### Questionnaire design

The questionnaire used in the present study was modified from a previous questionnaire to assess undergraduate dental students’ perceptions of IPE [15]. The previous 12 close-ended questionnaire items were extended and modified. As a result, the present questionnaire consisted of 20 close-ended questions and 2 open-ended questions to provide wider perceptions of IPE. The 20 closed-ended questions were further categorised into three domains, encompassing the effectiveness, preference, and importance of IPE. Each closed-ended question featured a five-point Likert scale, with response options ranging from “strongly agree” to “strongly disagree”. The scores distribution was as follow: strongly agree = 5, agree = 4, neutral = 3, disagree = 2, and strongly disagree = 1. The open-ended questions were: (1). “What factors will motivate you to participate in IPE learning activities?” and (2). “What barriers will discourage you from participating in IPE learning activities?”. Content validation of the questionnaire was performed by two experts (health profession educators) who had prior knowledge and experience in conducting IPE activities and questionnaire-based education research. They were invited to comment on any aspects of the questionnaire, including whether the items were reflective of the domains, whether any questions should be added to the domains and whether the terms used in the questionnaire were clear to students.

### Data analysis

The data analysis of the current study involved three tiers: psychometric validation of the modified questionnaire, descriptive and inferential statistics of quantitative data, and content analysis of qualitative data. First, the data were analysed using the IBS Statistical Package for the Social Sciences (SPSS) for Windows, Version 27.0, to assess the psychometric properties of the modified questionnaire. Different sources provide varying recommendations for the optimal sample size required for psychometric validation [17], but it is widely recommended that the minimum sample size should be at least 200, with a best practice ratio of 1 item to 20 respondents [18].

The data adhered to the assumptions of this confirmatory factor analysis, which included a large sample size and multivariate normal distribution of variables [19]. The model fit was evaluated based on the following statistics and indices: (a) factor loading values above 0.70 are preferred to explain the structure [20]; (b) the minimum discrepancy per degree of freedom (CMIN/df) should be less than 3 [21]; (c) the normed fit index (NFI), relative fit index (RFI), incremental fit index (IFI), Tucker Lewis index (TLI), and the comparative fit index (CFI) should be greater than 0.90 [22]; (d) the Root Mean Square Error of Approximation (RMSEA) should be less than 0.05 [23]. Subsequently, reliability values were determined to assess the composite reliability (CR) of the items. A CR value above 0.70 is considered acceptable. Next, the Average Variance Extracted (AVE) was calculated to establish the convergent validity of the instrument. Convergent validity is considered acceptable when (a) the AVE value exceeds 0.5 [24]; and (b) CR should be larger than AVE for each factor. Finally, to evaluate discriminant validity, correlation coefficients were calculated between the factors constituting the instrument. To ensure the discriminant validity of a model, the following are assessed: (a) Fornell-Larker criterion suggests that the latent variables square root of AVE must be larger than the correlation of that variable with other latent variables [24]; (b) the Heterotrait-Monotrait ratio (HTMT) suggests a criterion of 0.90 [25]; and (c) maximum shared squared variation (MSV) should be less than AVE [26].

Second, the data were analysed using the IBM SPSS for Windows, Version 29.0. (Armonk, NY: IBM Corp., USA). Descriptive statistics were used to provide an overview of the demographic characteristics. Subsequently, both simple and multivariable linear regression analyses, using variable identified as significant in the simple regression, were carried out to identify the factors that significantly influencing perceptions. Independent t-tests were conducted to investigate the differences in the domain and overall scores among variables that showed significance in the multivariable regression. The significance level was set at *p* < 0.05. Moreover, content analysis was performed for open-ended responses in the questionnaire. First, two analysts (GSSL & MHMH) used NVIVO 12 software to construct the initial codes. The initial codes were used as a guide for further coding until no additional code could be found from the respondents’ feedback. This was followed by refining and labelling the codes into different categories. Any coding disputes were discussed with the third analyst (YSN) until a consensus was obtained. The three analysts refined and approved all the final codes.

## Results

**Fig. 1:**
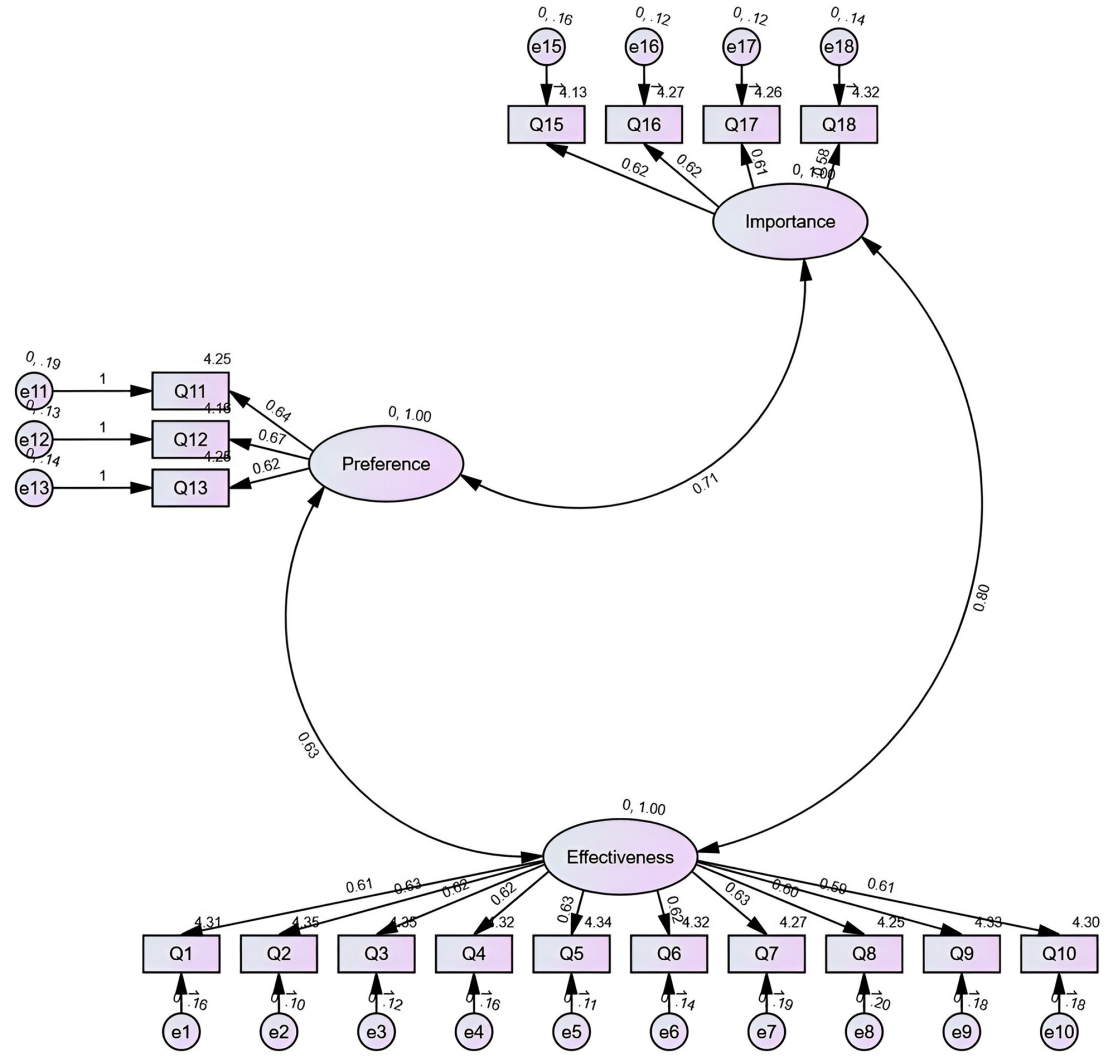
The three-factor model of the questionnaire items

A total of 397 undergraduate dental students completed all the 20-item questionnaire, resulting in a ratio of approximately one item per every 20 respondents. This ratio implies that the sample size was sufficient for the psychometric validation of the questionnaire. Approximately three-quarters of the participants were female (73.3%), belonged to the Malay ethnic group (56.9%), and enrolled in the clinical phase of their dental education (64.5%). These demographic details are summarised in Table 1. Figure 1 depicts the three-factor model, and items 14, 19, and 20 were removed due to their factor loadings lesser than 0.50. The remaining items have standardised regression weights ranging from 0.80 to 0.90, as shown in Fig. 1. The goodness-of-fit statistics for the model were CMIN/df = 2.27, NFI = 0.96, RFI = 0.95, IFI = 0.97, TLI = 0.97, CFI = 0.98, RMSEA = 0.06, and SRMR = 0.26, indicating satisfactory construct validity. Regarding reliability, the CR for the three factors, effectiveness, preference, and importance, were 0.96, 0.89, and 0.92, respectively, indicating satisfactory construct reliability. For convergent validity, the AVE for the three factors were 0.71, 0.73, and 0.74, respectively, and the CR values for each factor were larger than their respective AVE values as shown in Table 2. This demonstrated satisfactory convergent validity. With regards to discriminant validity, the square roots of AVE were larger than the intercorrelation between the factors as shown in Table 2. All MSV values were smaller than AVE. Moreover, the HTMT ratios, as presented in Table 2, were below 0.90, confirming the fulfilment of criteria for sufficient discriminant validity. Thus, the three-factor model (with the remaining 17 close-ended questionnaire items) demonstrated satisfactory discriminant validity and reliability.

**Table 1:** Respondent demographics (*n* = 397)

Variable	*n* (%)
**Gender**	
Female	291 (73.3)
Male	106 (26.7)
**Ethnicity**	
Malay	226 (56.9)
Chinese	130 (32.7)
Indian	24 (6.0)
Others	17 (4.3)
**University**	
AIMST	58 (14.6)
IIUM	67 (16.9)
UM	123 (31.0)
USM	149 (37.5)
**Phase of study**	
Preclinical	141 (35.5)
Clinical	256 (64.5)

AIMST: Asian Institute of Medicine, Science and Technology University, IIUM: International Islamic University Malaysia, UM: Universiti Malaya, USM: Universiti Sains Malaysia

**Table 2:** Analysis of CR, AVE, square root of AVE, and intercorrelation between the factors as well as the HTMT analysis

	CR	AVE	MSV	Effectiveness	Preference	Importance
Effectiveness	0.96	0.71	0.647	0.84	-	-
Preference	0.89	0.73	0.499	0.63*	0.86	-
Importance	0.92	0.74	0.647	0.81*	0.71*	0.86
**HTMT analysis**						
Effectiveness				-	-	-
Preference				0.598	-	-
Importance				0.761	0.641	-

*p < 0.001. CR = composite reliability, AVE = average variance extracted, MSV = maximum shared squared variation, HTMT = Heterotrait-Monotrait ratio

Undergraduate dental students generally expressed positive perceptions across various domains, with mean scores ranging from 4.13 to 4.35 (Table 3). The first domain entails Questions 1 to 10 which evaluated the students’ perception of the effectiveness of IPE. Questions 2 and 3 received the highest mean scores with 88.7% of the students agreeing that IPE improves their understanding of the roles and responsibilities of other healthcare professions, and 89.2% of them agreeing that IPE improves communication among students from various healthcare professions. The second domain (Question 11 to Question 13) evaluated students’ preference of IPE. The highest mean score was noted in Question 13 with 85.7% of the students agreeing that they feel comfortable to participate in learning activities (lectures, group discussions, seminars, clinical rotation etc.) related to IPE. Meanwhile, the third domain (Question 15 to Question 20) evaluated students’ perception of the importance of IPE. Question 18 received the highest mean score with 88.2% of the students agreeing that IPE is important to prepare students to work with other healthcare professions in providing effective treatment outcomes. Furthermore, the lowest mean score among all domains was noted in Question 15 with only 76.8% agreeing that it is important for universities to incorporate IPE as one of the educational goals.

**Table 3:** Means and standard deviations of students’ responses to each questionnaire item

		Mean (SD)
**Effectiveness of IPE**		
1.	IPE improves students’ understanding of the healthcare system	4.31 (0.73)
2.	IPE improves students’ understanding of the roles and responsibilities of other healthcare professions	4.35 (0.71)
3.	IPE improves communication among students from various healthcare professions	4.35 (0.71)
4.	IPE allows students to respect and trust students from other healthcare professions	4.31 (0.74)
5.	IPE improves collaborative practice among students from various healthcare professions	4.34 (0.71)
6.	IPE improves students’ understanding of the limitations of their field within the healthcare system	4.32 (0.73)
7.	IPE improves problem-solving skills among students from various healthcare professions	4.27 (0.77)
8.	IPE reduces students’ misperceptions about other healthcare professions	4.25 (0.75)
9.	IPE improves quality of patient care	4.32 (0.73)
10.	IPE improves students’ confidence to learn together with other healthcare professional students	4.30 (0.74)
**Preference of IPE**		
11.	I feel comfortable to attend learning activities that are taught jointly by lecturers from other healthcare professions	4.24 (0.78)
12.	I feel comfortable to attend learning activities with students from other healthcare professions	4.16 (0.77)
13.	I feel comfortable to participate in learning activities (lectures, group discussions, seminars, clinical rotation etc.) related to IPE	4.25 (0.73)
14.	I do not feel comfortable for IPE to be incorporated into my studies	Removed
**Importance of IPE**		
15.	It is important for universities to incorporate IPE as one of the educational goals	4.13 (0.74)
16.	It is important for universities to provide opportunities for students to participate in IPE	4.27 (0.71)
17.	IPE is important to prepare students to understand scopes of practice of other healthcare professions	4.26 (0.70)
18.	IPE is important to prepare students to work with other healthcare professions in providing effective treatment outcomes	4.31 (0.70)
19.	It is not important for students to learn together with students from other healthcare professions students	Removed
20.	IPE is not important for students to improve their teamwork skills.	Removed
**Open-ended question**		
21.	What factors will motivate you to participate in IPE learning activities?	
22.	What barriers will discourage you from participating in IPE learning activities?	

SD = standard deviation

Table 4 reveals the factors associated with perceptions of interprofessional education among the respondents. Gender, ethnicity, particularly the Indian ethnic group, and the respondents’ clinical phase (phase of the study) demonstrated significant relationships with perceptions at the simple regression level. Notably, the Indian group was significant in simple regression but, being only a small proportion of the total sample’s ethnicity, it was not included in the multivariable analysis. In the multivariable linear regression analysis, gender and clinical phase remained statistically significant. Male respondents scored significantly lower in total perception (coefficient: -2.0; 95% CI: -3.99, -0.01; *p* = 0.049) compared to their female counterparts. Furthermore, the stage of dental education significantly influenced perceptions, with clinical-phase respondents exhibiting less favourable views, as reflected by a negative adjusted regression coefficient of -3.23 (95% CI: -5.38, -1.08) and a *p*-value of 0.003, compared to preclinical-phase respondents. In contrast, neither ethnicity nor university affiliation significantly influenced the perceptions based on the multivariable analysis.

**Table 4:** Factors associated with perception scores (*n* = 397)

Variables	SLR^a^		MLR^b^		
	b^†^ (95% CI)	*p*-value	Adj. b^◊^ (95% CI)	t*-*stat.	*p*-value
Gender					
Female	Reference			Reference	
Male	-3.53 (-5.70, -1.36)	0.001	-2.0 (-3.99,-0.01)	-2.95	0.049
Ethnicity					
Malay	Reference				
Chinese	-1.12 (-3.19, 0.95)	0.288	-	-	-
Indian	6.14 (2.11, 10.18)	0.003	-	-	-
Others	0.49 (-4.31, 5.29)	0.842	-	-	-
University					
AIMST	Reference				
IIUM	0.40 (-2.19, 3.0)	0.759	-	-	-
UM	1.27 (-0.83, 3.36)	0.236	-	-	-
USM	-1.95 (-3.95, 0.05)	0.056	-	-	-
Clinical phase					
Preclinical	Reference			Reference	
Clinical	-2.14 (-4.16, -0.12)	0.038	-3.23(-5.38,-1.08)	-1.98	0.003

SLR = Simple linear regression

MLR = Multivariable linear regression (The model fits reasonably well; model assumptions are met. There is no interaction between independent variables and no multicollinearity problem exists)

^†^ Crude regression coefficient

^◊^ Adjusted regression coefficient

AIMST = Asian Institute of Medicine, Science and Technology University, IIUM = International Islamic University of Malaysia, UM = University of Malaya, USM = Universiti Sains Malaysia

Given that gender and clinical phase were the only significant variables in the multivariable linear regression analysis, a comparative analysis was conducted to gain further insights into their impact on perception scores related to interprofessional education, as presented in Table 5. Gender played a pivotal role in shaping perceptions, with female respondents consistently demonstrating higher mean scores in all domains (“Effectiveness”: *p* = 0.001, “Preference”: *p* = 0.010, “Importance”: *p* = 0.023), and they also scored higher in the overall domain (*p* = 0.001). On the other hand, the clinical phase emerged as a significant factor solely in the “Effectiveness” domain, with preclinical-phase respondents scoring higher than their clinical-phase counterparts (*p* = 0.026). Notably, preclinical-phase students also exhibited a significantly higher mean overall score (*p* = 0.038).

**Table 5:** Comparative analysis of perception scores by gender and clinical phase

Domain	Gender			Phase of Study		
	Female (*n* = 291) Mean (SD)	Male (*n* = 106) Mean (SD)	*p*-value ^a^	Preclinical (*n* = 141) Mean (SD)	Clinical (*n* = 2) Mean (SD)	*p*-value ^a^
Effectiveness	43.7 (6.01)	41.5 (6.73)	0.001	44.1 (5.0)	42.6 (6.39)	0.026
Preference	12.8 (2.0)	12.2 (2.15)	0.010	12.9 (1.99)	12.52 (2.09)	0.081
Importance	17.2 (2.48)	16.5 (2.66)	0.023	17.2 (2.32)	16.9 (2.66)	0.251^b^
Overall	73.7 (9.47)	70.2 (10.39)	0.001	74.2 (9.42)	72.0 (9.99)	0.038

SD = standard deviation, ^a^ = Independent *t*-test, ^b^ = equal variances not assumed

Possible maximum scores: Effectiveness: 50, Preference: 15, Importance: 20, Overall: 85

Open-ended responses revealed possible factors motivating participation in IPE learning activities (Table 6). Students expressed a keen interest in the integration of diverse perspectives and acquiring knowledge beyond the confines of their discipline. Examples of responses were as follows:

**Table 6:** Identified codes related to motivations and barriers along with the frequency of mentions by the respondents

Code	Frequency
**Motivations**	
Learning from different professionals	15 times
Understanding the healthcare system	11 times
Integration of different professions	13 times
Broadening perspectives	21 times
Teamwork enhances patient care	25 times
**Barriers**	
Packed schedules	31 times
Coordination difficulties	15 times
Lack of understanding of IPE	22 times
Professional stereotypes	19 times
Unclear learning goals/outcomes	10 times

*Can have the opportunity to learn from different healthcare professionals.*

*Learning other professions can improve the understanding of the entire healthcare system.*

*Able to integrate different professions into one.*

*Discussing with other healthcare students will broaden one’s perspectives.*

Furthermore, students underscored the significance of teamwork in enhancing patient care as a key motivator. Examples of students’ responses included:

*Can improve teamwork skills with other students from different faculties.*

*Promotes teamwork and improves patient care.*

*Increase the cooperation and teamwork among healthcare workers.*

On the other hand, the barriers to participating in IPE learning activities included tightly packed schedules, lack of understanding regarding IPE and misconceptions towards dental education (Table 6). Some students highlighted:

*Packed schedule due to lectures and labs.*

*The need to sort out a time for all (students) to gather for discussion.*

*Less understanding on how IPE actually works.*

*Unclear with the actual learning outcomes and goals of IPE.*

*Stereotypes among different professions will exist.*

*There are always misconceptions that dentistry is not as important as medicine.*

Additionally, some students expressed nervousness and fear to participate in IPE activities. Examples of student responses in this regard included:

*Nervous to accept and adapt IPE.*

*Scared to say something which is incorrect or wrong.*

*May feel insecure when having discussions with students from other professions.*

## Discussion

The present multi-institutional study sheds light on the perceptions of Malaysian undergraduate dental students regarding IPE. Prior to the presentation of findings, psychometric validation was conducted to affirm the validity and reliability of the adapted questionnaire items. Notably, the consistently high mean scores (> 4.00/5.00) across all domains reflect the students’ favourable perceptions, underscoring the robustness of their positive views towards IPE. Based on these findings, most students agreed that IPE enhances their comprehension of roles and responsibilities in various healthcare professions, aligning with prior studies on collaborative learning with dental hygiene and oral health therapy students [27, 28]. Recognising contributions from other healthcare professionals such as dental technologists, medical physicians, nurses, and pharmacists enables seamless teamwork among dental professionals, ensuring holistic and well-coordinated patient treatment plans [29, 30]. This understanding of roles and responsibilities also cultivates an awareness of the broader healthcare landscape, prompting dental professionals to consider an integrated, patient-centred approach to healthcare [28]. Moreover, the present findings resonate with IPE’s foundational principles, emphasising collaborative learning and fostering a comprehensive understanding of diverse professional roles and responsibilities within a multi-professional healthcare team [12].

Similarly, most students acknowledged that IPE improved communication among students from various healthcare professions which is in accordance with other similar studies [30–32]. These findings emphasise IPE’s potential to foster interpersonal skills and overcome communication barriers among future healthcare professionals [28, 31]. However, it is noteworthy that approximately one-third of students did not agree with the notion that IPE would effectively diminish misconceptions about various healthcare professions. This sentiment was evident in the open-ended responses, where students asserted the persistence of stereotypes among different professions and the prevailing misconception that dentistry is less important than medicine. This prompts further exploration into the specific aspects or challenges that may contribute to such perceptions. Professional hierarchies and preconceptions have been shown to influence IPE learning sessions, potentially impeding communication, and interaction among all participants [32–34]. Addressing and understanding these concerns can inform future enhancements to IPE programs, ensuring a more comprehensive and impactful educational experience.

Although students generally agreed that they feel comfortable to participate in various IPE-related learning activities, a low mean score was noted when examining students’ comfort levels in attending learning activities with students from other healthcare professions. This divergence in responses indicates the necessity of a sophisticated strategy for creating IPE programs that tackle students’ discomfort or fear while working with peers from different healthcare professions [35]. In line with the open-ended responses, nervousness and fear were expressed by some participants. This highlights that emotional obstacles may prevent students’ engagement in IPE activities which was also reported in other studies [32, 35]. This reluctance may stem from the established fragmented curricula, where students are comfortable with their peers and resist integrating new students. Furthermore, as IPE is still considered new to most of our participants, they may lack the readiness to shift from traditional uni-disciplined teaching approaches. Hence, it is imperative to create a supportive and inclusive learning environment that encourages open communication and values the contributions of all participants [4]. Surprisingly, the lowest mean score was noted among students regarding the importance of incorporating IPE into universities’ educational goals. This could probably be due to the existing curriculum which separates dentistry and medicine, limiting exposure and opportunities for IPE. In addition, the solitary nature of dentistry may contribute to the perception that IPE may not be necessary in the dental curricula [36]. Further exploration is warranted to understand the factors influencing this perception, considering potential institutional-level barriers or challenges in implementing IPE.

The importance of teamwork in enhancing patient care has emerged as another significant motivator for students [32]. The recognition that collaboration across various healthcare professions contributes to improved teamwork skills and ultimately enhances patient care underscores the practical implications of IPE [13]. Conversely, participants also highlighted tightly packed schedules as a challenge for IPE. Given that IPE requires accommodating students from various health professions in a shared setting, inflexible and conventionally packed schedules limit rooms for IPE activities. Thus, flexible scheduling and effective time management strategies are essential considerations for designing and implementing successful IPE initiatives. The observation of lower total perception scores among male respondents compared to their female counterparts aligns with findings in various studies involving dental and health profession students [9, 37]. This gender-related trend implies that female students might exhibit greater openness and involvement in IPE initiatives, while male students may indicate a stronger influence by traditional gender roles in controlling professional knowledge and mechanisms of exclusion [38, 39]. Consequently, the gender gap in perception scores indicates a potential necessity for tailored interventions addressing specific challenges or concerns experienced by male students in IPE.

Furthermore, students in the clinical phase displayed less favourable perceptions, particularly in the “Effectiveness” domain, contradicting findings from other studies [9, 40]. Early engagement and networking foster mutual respect and diminish stereotypes by allowing students to participate in IPE without a firmly established “doctor professional identity”, potentially mitigating intergroup discrimination and addressing lower levels of prejudice [39]. Nevertheless, neither ethnicity nor university affiliation significantly influenced the perceptions. While Indian ethnic respondents appeared to hold favourable perceptions in the univariable analysis, caution is warranted due to the potentially under-represented sample size. This indicates that gender and clinical phase may exert a more substantial influence on IPE perceptions than ethnic background. Moreover, the lack of significance for university affiliation suggests that the perceived effectiveness, preference, and importance of IPE are not notably influenced by the specific educational institution as dental curricula across dental schools in Malaysia follow similar Program Learning Outcomes [41]. The consistent alignment in educational goals and learning outcomes may contribute to a uniform understanding of IPE among students irrespective of their university affiliation.

The present study exhibited several strengths, including the improvement of an existing questionnaire through content and psychometric validation, thereby establishing a reliable tool for assessing perceptions of IPE. This validated questionnaire can be used by future researchers to continue enriching the literature on IPE. Next, the qualitative analysis benefited from a triangulation approach among analysts, reinforcing the credibility of the study’s findings. Nevertheless, certain limitations should be acknowledged. First, the study only involved four dental institutions in Malaysia, limiting its generalisability. Second, the success of IPE requires engagement from different professions; hence, the validated questionnaire may be used in future studies to assess and compare perceptions of IPE across different professions, such as medicine, nursing, and other allied health professions. These comparative insights would provide a foundational understanding of diverse learning needs, informing strategies to promote active participation in IPE among health profession students. The goal of incorporating IPE into curricula is to foster an interprofessional mindset among students before they graduated [42]. It could be challenging to provide these learning opportunities, but having a university-based central IPE system, effective team representation from all healthcare faculties, extensive curriculum mapping, and modification of the existing teaching methods would be the solution [43, 44]. In addition, optimising effective IPE programs requires addressing identified motivations and barriers, alongside implementing strategies to increase students’ comfort in collaborating with diverse healthcare professions, fostering inclusivity and a harmonious learning environment.

## Conclusion

The present study provides valuable insights into the perceptions of IPE among undergraduate dental students in Malaysia. It reveals a generally positive perception towards IPE, with a notable emphasis on its ability to improve understanding and communication among diverse healthcare professionals. Nevertheless, significant relationships were noted between gender and clinical phase with perceptions of IPE, whereby female students and those in the preclinical phase expressed more favourable perceptions. Despite generally favourable responses, challenges such as tightly packed schedules, lack of understanding, and concerns about fitting into IPE activities were identified as barriers to participation. The results underscore the importance of strategic planning in transitioning from traditional education to IPE in dental curricula, particularly in resource-constrained developing countries, such as Malaysia. Addressing logistical challenges, improving awareness, and creating a supportive and inclusive learning environment are crucial for fostering dental students’ active participation in IPE activities. As the healthcare landscape continues to evolve, embracing IPE becomes imperative for dental practitioners to develop essential skills and contribute effectively to collaborative team-based patient care, ultimately improving overall healthcare outcomes.

## Data Availability

The raw data that support the findings of this study are available from the corresponding author upon reasonable request.

## Abbreviations

AVE: Average Variance Extracted
BDS: Bachelor of Dental Surgery
CMIN/df: Minimum Discrepancy per Degree of Freedom
CFI: Comparative Fit Index
CR: Composite Reliability
DDS: Doctor of Dental Surgery
HTMT: Heterotrait-Monotrait ratio
IFI: Incremental Fit Index
IPE: Interprofessional Education
IPEC: Interprofessional Education Collaboration
MSV: Maximum Shared Squared Variation
NFI: Normed Fit Index
RMSEA: Root Mean Square Error of Approximation
RFI: Relative Fit Index
SPSS: Statistical Package for the Social Sciences
TLI: Tucker Lewis Index
